# Analysing the evolution of computer science events leveraging a scholarly knowledge graph: a scientometrics study of top-ranked events in the past decade

**DOI:** 10.1007/s11192-021-04072-0

**Published:** 2021-07-10

**Authors:** Arthur Lackner, Said Fathalla, Mojtaba Nayyeri, Andreas Behrend, Rainer Manthey, Sören Auer, Jens Lehmann, Sahar Vahdati

**Affiliations:** 1 University of Bonn, Bonn, Germany; 2 Faculty of Science, University of Alexandria, Alexandria, Egypt; 3 Nature-Inspired Machine Intelligence, Institute for Applied Informatics (InfAI), Dresden, Germany; 4 Institute for Telecommunications (INT), TH Köln, Köln, Germany; 5 NetMedia Department, Fraunhofer IAIS, Dresden, Germany; 6 L3S Research Center, University of Hannover, Hannover, Germany; 7 TIB Leibniz Information Centre for Science and Technology, Hannover, Germany

**Keywords:** Scientific Events, Ontology, Metadata Analysis, Scholarly Communication, Metric Suite

## Abstract

The publish or perish culture of scholarly communication results in quality and relevance to be are subordinate to quantity. Scientific events such as conferences play an important role in scholarly communication and knowledge exchange. Researchers in many fields, such as computer science, often need to search for events to publish their research results, establish connections for collaborations with other researchers and stay up to date with recent works. Researchers need to have a meta-research understanding of the quality of scientific events to publish in high-quality venues. However, there are many diverse and complex criteria to be explored for the evaluation of events. Thus, finding events with quality-related criteria becomes a time-consuming task for researchers and often results in an experience-based subjective evaluation. OpenResearch.org is a crowd-sourcing platform that provides features to explore previous and upcoming events of computer science, based on a knowledge graph. In this paper, we devise an ontology representing scientific events metadata. Furthermore, we introduce an analytical study of the evolution of Computer Science events leveraging the OpenResearch.org knowledge graph. We identify common characteristics of these events, formalize them, and combine them as a group of metrics. These metrics can be used by potential authors to identify high-quality events. On top of the improved ontology, we analyzed the metadata of renowned conferences in various computer science communities, such as VLDB, ISWC, ESWC, WIMS, and SEMANTiCS, in order to inspect their potential as event metrics.

## Introduction

Scientific communication is intended as a knowledge exchange ecosystem. Scholars disseminate their research results by publishing written documents. This way of communication has developed over time and consists of certain steps and involves corresponding stakeholders such as publishers, authors, reviewers, and organizers. Institutions, research groups, and researchers find themselves in a competitive scholarly communication system. In recent years, scholarly communication has faced rapid changes leading to the production of a large volume of scholarly artifacts that have become easily accessible Priem (2013). Publishing via scientific events such as conferences and workshops is one of the main channels for disseminating research results for certain scholarly communities. Scientific events are also considered as the main target for researchers who want to connect with other community members and stay informed about their topics of interest. In today’s scholarly communication, the career of scholars generally depends on the extent to which their success is recognized by the community.

Due to the often subjective nature of the concept of *quality* in research, there exist several definitions by different researchers. Quality is defined as excellence, value, conformance to specifications, or meeting user expectations Kahn et al. (2002). More generally, it is widely accepted as *fitness for use* Juran (1974); Knight and Burn (2005) which we follow in this research work as well. Application of this meaning on the domain of scholarly communication reflects the extent to which the totality of features and characteristics of an artifact lead to a successful fulfillment of scholar’s needs. The cumulative nature of scientific knowledge necessitates the quality assessment of artifacts and involved agents, organizations, and events particularly important for scholarly communication. The quality of scholarly artifacts and other elements of scholarly communication, such as events, has multiple characteristics. Researchers combine assessments of these characteristics in different ways depending on their view or task. For researchers, upcoming events on a specific topic can be interesting concerning the closeness of the location, the validity of the publisher, and the reputation of speakers and organizers. Another researcher may only focus on the reputation of the events with respect to their acceptance rate. Therefore, depending on the incentive and objectives of the individual researchers or communities, there is a wide range of requirements and needs in the context of the scholarly communication domain. Particularly, the question of how to assess the quality of a scientific event has been discussed recently in the context of “predatory conferences”.

While each research community has its own formal and informal rules for quality standards, individual researchers often significant challenges regarding determining scholarly communication related queries such as finding a matching target event to submit their research results. To the best of our knowledge, currently available services for scientific event exploration offer only an overview of existing and upcoming events. Furthermore, data about scientific events is often unstructured and not well preserved for further uses. In addition, such information is spread across numerous platforms with different standards. Therefore, comprehensively organizing scholarly event metadata has the potential to answer meta-research queries such as identifying current research topics and future trends, finding experts on specific research, estimating the cost and efforts of planning an event.

The research presented in this article aims to conceptualize a particular area of scholarly communication via events and all related entities, such as stakeholders of scientific events, and prototype the semantic and systematic answering of such queries. In this work, we use OpenResearch.org (OR)^1^, a wiki-based crowd-sourcing platform, to collect and curate scholarly event metadata in a structured format. With a focus on particular areas of scholarly communication in ontology development and extension of Openresearch.org, the following research questions are addressed:

- * RQ1: Can we represent scientific event metadata using a semantic representation aiming at supporting answering meta-research queries?*
- *RQ2: What are the main characteristics of renowned scientific events in computer science*?
- *RQ3: Can we develop a service on top of semantically represented data of scientific events to support scholarly communication*?

By answering these questions we show that the application of metadata allows for an objective evaluation of the quality of scientific events and the observation of trends and quality-related changes over time. We present how enriched metadata together with the proposed metrics can be successfully employed by researchers in order to compare events and find the most relevant ones for disseminating their scientific results.

This article is structured as follows: “Related work” provides a summary of related work. In “Motivating example” a motivating example for a meta-research query about scholarly events is presented. Description of the domain conceptualization and ontology extension of Openresearch.org is represented in “Domain conceptualization”. A list of sample analyses using semantically represented metadata of scientific events is shown in “Events metadata collection and analysis”. In “Semantic mediaWiki platform”, we provide a short description of the Openresearch.org platform and we conclude the work in “Conclusion and future work”.

## Related work

Metadata analyses of scientific events have received much attention in the past decade due to the mega-trend of digitization and the ease of scientific events organization. Several efforts have been made for assessing or tracking the evolution of a specific scientific community by analyzing the metadata of particular event series Aumüller and Rahm (2011); Barbosa et al. (2017); Fathalla and Lange (2018); Biryukov and Dong (2010); Fathalla et al. (2017, 2018); Vahdati et al. (2016); Nayyeri et al. (2020). Currently, there are several single sources on scientific events and source-dedicated services available for researchers to explore events and as a channel for event organizers to disseminate information about their event. Biryukov and Dong Biryukov and Dong (2010) investigated collaboration patterns within a research community using information about authors, publications, and conferences. Similarly, David and Rahm Aumüller and Rahm (2011) analyzed affiliations of database publications using author information from DBLP, and Nascimento et al. (2003) analyzed the co-authorship graph of SIGMOD conference publications. Singh et al. Singh et al. (2016) proposed a framework, ConfAssist, to identify whether a conference is top-tier or not. They identified various features related to the stability of conferences that might help to separate a top-tier conference from the non-top-tier ones. Fathalla et al. Fathalla et al. (2019) published a 5-star dataset (EVENTSKG) of top-ranked computer science events. EVENTSKG contains metadata of 73 event series using the Scientific Events Ontology Fathalla et al. (2019) as a reference ontology for describing events metadata.

In addition to scholarly event metadata analysis, there are event metadata management platforms. *CFP Manager*Issertial and Tsuji (2015) is a domain-specific tool to extract metadata of events from an unstructured text representation of CFPs. This tool is designed as a plug-in to other services and specific for computer science call for papers. *Cfplist*^2^ works similarly to WikiCFP but focuses on social science-related subjects. *SemanticScholar*^3^ offers a keyword-based search facility that shows metadata about publications and authors. It uses artificial intelligence methods in the back-end and retrieves results based on highly relevant hits with the possibility of filtering. *Conference.city*^4^ is a new service initialized in 2016 that lists upcoming conferences by location. For each conference, title, date, deadline, location, and the number of views (of its page in conference.city) are shown. *PapersInvited*^5^ focuses on collecting CfPs from event organizers and attracting potential participants.

Similar to call for papers, there are databases and bibliographic indices for event proceedings that are available for the community free of charge. *DBLP* “Computer Science Bibliography”^6^ is a free well-known bibliography database that store events proceedings as well as events metadata, such as subevents and location. *ACM Digital Library* stores full-text articles and e-books published by the ACM as well as bibliographic literature covering computing and information technology, including proceedings.^7^ Similar services are provided by other proceeding publishers as *Scopus*^8^ by Elsevier or *IEEE Xplore*^9^ by the Institute of Electrical and Electronics Engineers. SpringerNature takes one step further and provides a SciGraph interface for their publications.^10^

The Springer LOD^11^ provides a dataset about conference proceedings—published by this publisher, e.g., in the Lecture Notes in Computer Science series—for public reuse. However, the number of the considered event properties is limited to the basic metrics such as event title, date, location, and this dataset does not adequately cover quality-related properties. Similarly, *ScholarlyData*^12^ provides RDF dumps for scientific events Nuzzolese et al. (2016). Conference-Ontology, a new data model developed for ScholarlyData, improves over already existing ontologies about scientific events such as the Semantic Web Dog Food (SWDF) Nuzzolese et al. (2016) ontology. An analysis of a set of 110 conferences metadata has been performed to conform to the proposed hypothesis. Several studies, for example Fathalla et al. Fathalla et al. (2017, 2018) and Hiemstra et al. Hiemstra et al. (2007), have been conducted on analyzing different computer science communities using the metadata of several event series, while Barbosa et al. Barbosa et al. (2017) have analyzed full papers published in the Brazilian Symposium on Human Factors in Computing Systems (IHC) conference series in the period 1998–2015. In 2020, Fathalla et al. Fathalla et al. (2020) have extended their analysis of computer science events metadata to involve scientific events belonging to four fields of science, namely Computer Science, Physics, Engineering, and Mathematics.

A key problem not sufficiently addressed in much of the literature is that the characteristics of top-ranked scientific events are not well identified and analyzed. Accordingly, in this study we utilize Semantic Web technologies (i.e., RDF, OWL and SPARQL) in order to support smart data analytics of scientific events metadata by producing a scholarly Knowledge Graph of Computer Science events.

## Motivating example

**Fig. 1:**
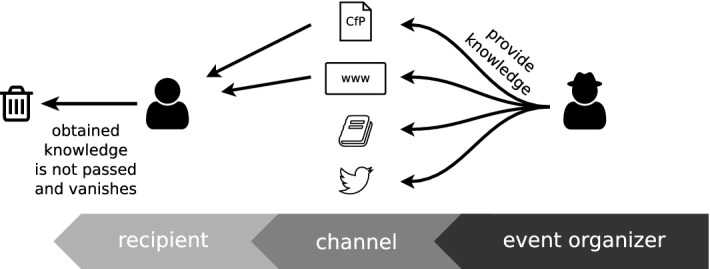
Information flow from event organizers (right-hand side) to the interested audience (left-hand side). *Amanda* obtains only from two of the channels, the event organizers have provided

In this section, we provide an example to motivate the problem of the difficulty in finding appropriate scientific events (regarding certain criteria) for publishing research results. We show an example of discovering a potential list of scientific events within a certain community. Possible types of stakeholders among researchers are either event organizers, authors, reviewers, sponsors, speakers, and participants, etc. Finding the right scientific events is crucial from the roles and parties point of view, however, this can only be developed over time by the researchers themselves which requires time and experience and is prone to omissions. Therefore, it is helpful to have automatic methods that can ease the discovery of events considering quality with regards to a set of certain metrics. Let us consider a case where a researcher (e.g., Amanda) wants to determine events, satisfying certain criteria such as topic-relatedness, geographical restrictions, and time, in order to submit her work. One trivial way to solve this is to ask colleagues and read the call for papers (CfP) published in conference management services (popular ones are listed below), which is time-consuming and takes effort. For example, with these two sources (i.e., asking colleagues and reading CfP), he is only able to find the events that take place in Europe and are related to his field of interest. However, the call for papers of different events gives limited or no clues about the quality of the event, which can be reflected by the reputation of the organizers and keynote speakers, the values of sponsors, etc. Therefore, *Amanda* has to check events websites, previous related events and possibly has to read the proceedings, to obtain more information about these events. One key quality indicator of the scientific rigor of an event, the acceptance rate, for example, is in most cases only available from the preface of the proceedings. Now, the knowledge that is gathered/acquired by *Amanda* about events series is not accessible to others especially newcomers (cf. Fig. 1). To address this, we developed the service OpenResearch.org to curate and present event metadata in a structured format in order to make it publicly available as Linked Open Data (LOD) (more details in Sect. 6).

Several online services already now help researchers to keep track of information about upcoming conferences, workshops, meetings, seminars, events, and journals including:

- **WikiCFP**^13^ is a collection of CfPs, which can be searched by year and text match (e.g. search for “Germany” in 2018 and retrieve all CfPs which include “Germany” somewhere in the CfP). CfPs can be sorted by title, field, location and year.
- **CFP List**^14^ is a similar service but provides the users a map with markers for all upcoming events on the front page. A calendar widget lists the next dates for events and deadlines for paper submission. These visual tools make it easier for scientists to browse events.
- **Confsearch**^15^ is based on the data from DBLP^16^ and uses a wiki-principle for crowd-gathering metadata about conferences, like dates and homepage links. Search results are presented as a list with a calendar view to compare the event dates in the search result.
- **Conference.city**^17^ provides also metadata about conferences of other domains than computer science. Conferences can be filtered by topic, date, and continent. It also relies on user-generated content like *confsearch* which explicitly mention that it may include technical, typographical, or photographic errors.
- **AllConferences**^18^ is another index for conferences with different domains. It is a special conference search service, where organizers can pay to list their conference in the second or first tier of search results.

In summary, all these services have very limited and not sufficiently well structured metadata about scholarly events, in particular wrt. the scientific quality of the events.

## Domain conceptualization

In this section, we focus on the scientific communication domain, particularly, scientific events and all related entities, such as fundamental concepts, stakeholders of scientific events, scientific publications produced, and their spatial and temporal data.

**Fundamental Concepts** An event is a scientific gathering of scholars who are working on similar topics. Research results as articles are submitted to the events and accepted ones are presented. Scientific presentation talks accompanied by articles are the communication means of scientific events. Researchers submit their research results and those passing the review phase successfully are presented in the event. Registration for the event is one of the main activities. It is not sufficient to have an accepted work, scholars need to register for the events and it has its own process. Identity shows the ways the abstract concept of the event is presented to the scholarly communities. It can point to the event homepage, call for paper emails, etc.

**Scientific Events Stakeholders** A event stakeholder is a scholar involved in the scholarly communication chain during the organization and holding phase of the event, such as scientific chairs, other organizers, reviewers, participants, authors, speakers, etc, The audience attending an event, comprises attendees without having any presentation, aiming for networking and to keep up with the work in his field, Sponsors are the source of the financial support to the event to gain visibility in the communities targeted by the event. Organizing organizations comprises the institutes or universities which are hosting or organizing the event. Usually, this points to the affiliation of the main chairs.

**Spatial data** The data or information that identifies the geographic location of an event in terms of the hosting country, visited by that event is considered as geographically spatial data.

**Temporal data** The data that refers to the period of time, in terms of the months of the year, each year in which an event takes place is considered as temporal data.

**Fig. 2:**
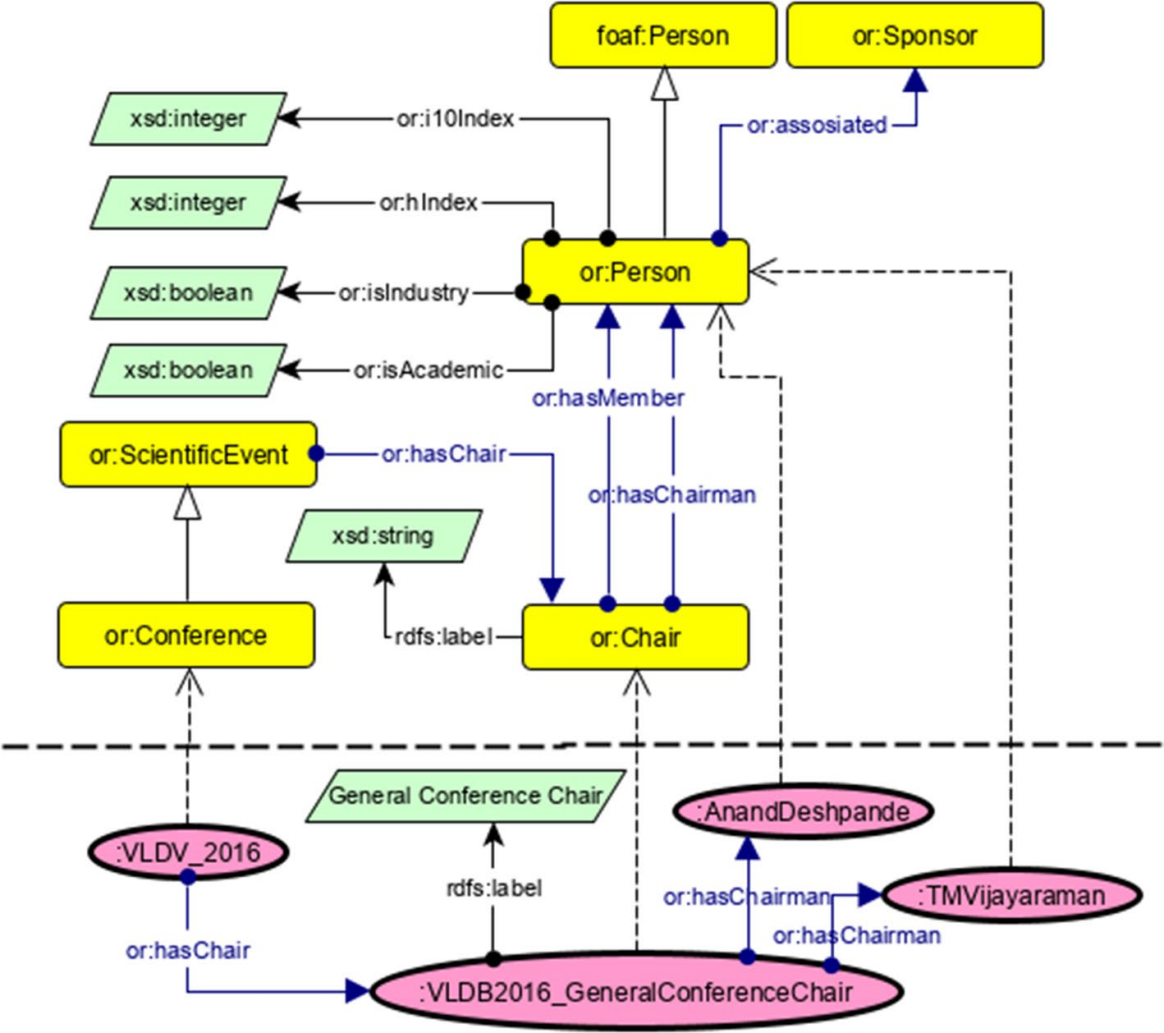
Definition of or:Chair. Upper part: the TBox is shown with the general concept of or:Chair and its relations to other concepts. Lower part: the ABox is shown with a real world example from VLDB2016

We aim at providing a comprehensive, well-structured knowledge graph in order to provide more holistic exploration of events based on consistently structured metadata including scientific quality indicators, interlinking features and a query interface. This knowledge graph is organized using RDF statements as atomic constituents by utilizing the RDF, RDF Schema, and OWL standards. Here we describe the proposed knowledge graph from two different views:

1. *Taxonomy level* (also referred to as TBox), where we describe the classes and how a class implies several properties for all their instances, and
2. *Individual level* (also referred to as ABox), which shows concrete instances and their properties with values from the real world.

**Fig. 3:**
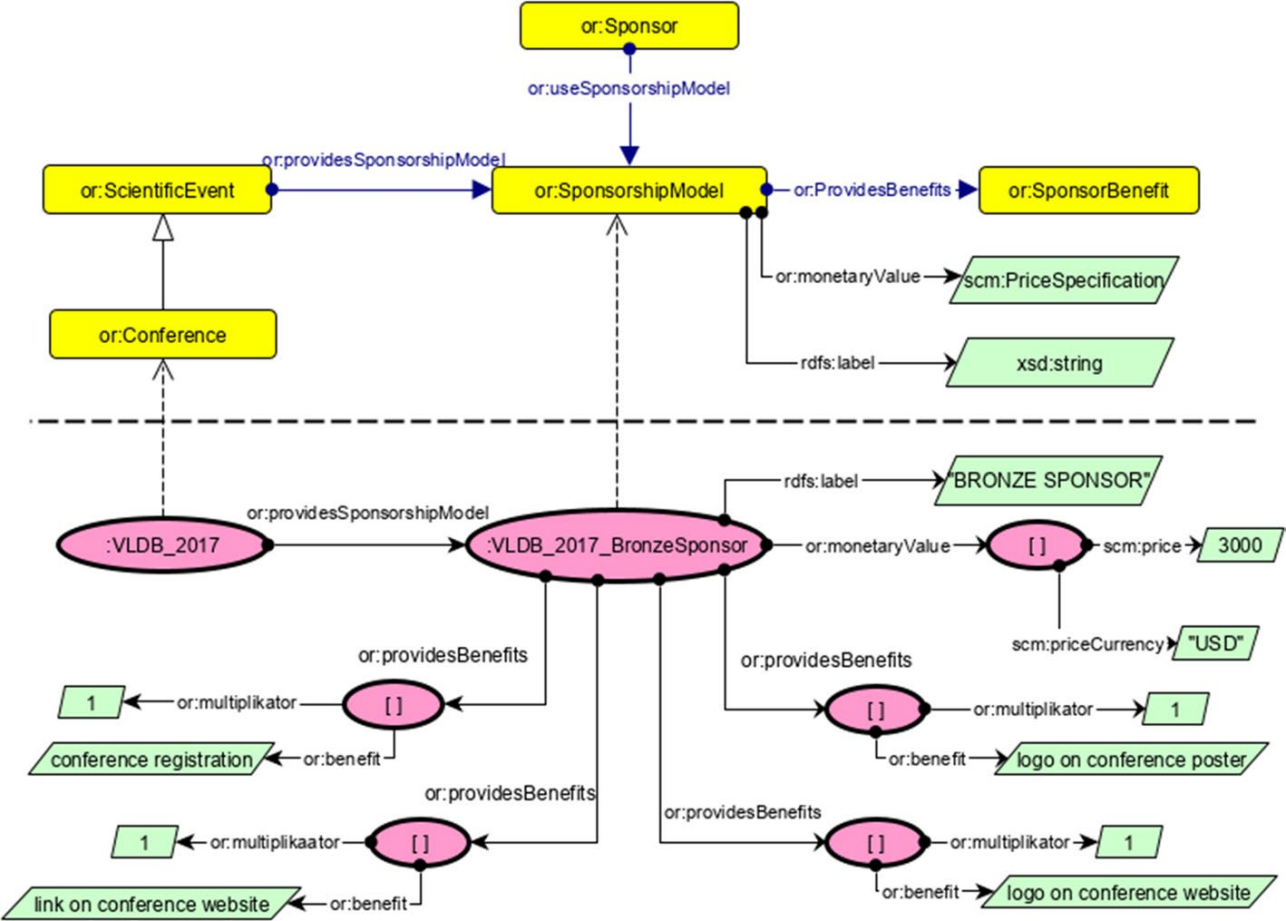
Representation of sponsorship model. Upper part: TBox is shown for the class or:SponsorshipModel and its relations to other concepts. Lower part: ABox is shown with a real world example from VLDB2017

**Fig. 4:**
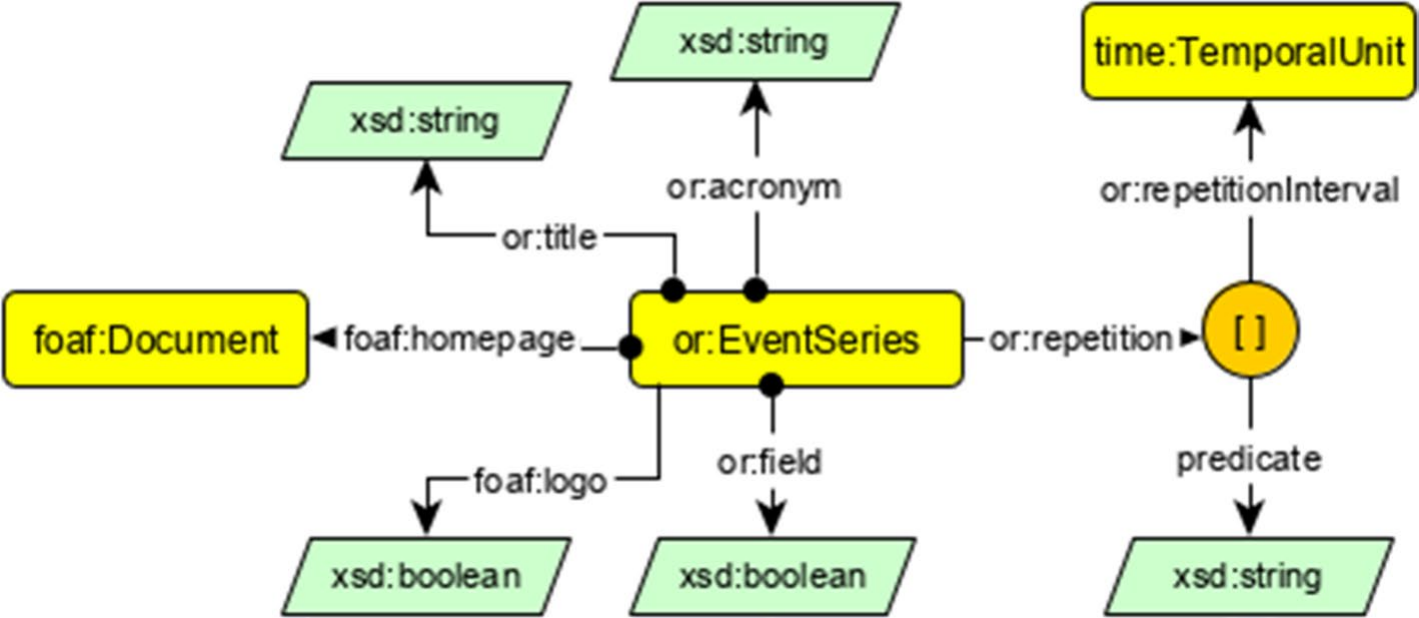
Ontology of scientific event series, with the information about their regularity and temporality. All event series keep a certain acronym unless it changes or good reasons

A list of core entities is considered in the ontology of Openresearch.org which we discuss here including information about their ontological description:

- *Events* are represented by the class or:ScientificEvents, for conferences and workshops, which also defines common properties for their description. Members of this class are supposed to have a start and end date, a location, a title and are organized by a group of one or more persons, i.e., chairs.
- *Persons* involved in the Domain of Scientific Events are represented by the class or:Person, which is a subclass of foaf:Person. or:Person has domain-specific properties from the scientific events domain to describe domain specific attributes of a scientist or organization associated person. Events are organized by one or more Chairs, which is represented by the class or:Chair, i.e., group of persons, which are responsible for organizing a specific scientific event. Members of this class are supposed to have or:hasChairman (i.e., the person who head the chairs) and or:hasMember (i.e., persons who work as a chair). Figure 2 shows these relations at the upper taxonomy level (TBox) and an employment at the bottom individual level (ABox).
- *Sponsors*, as further stakeholders of scientific events, are represented by the class or:Sponsor. Being a sponsor implies that an individual is using one or more of the sponsorship models or:SponsorshipModel, that a or:ScientificEvent provides. This relation is shown in Fig. 3. Members of or:SponsorshipModel class are supposed to have or:monetaryValue, the amount of money a sponsor has to give event organizers to get this sponsorship with all its benefits, and or:providesBenefits, points to one benefit with a multiplier, e.g., a blank node with the multiplier 3 (in Fig. 3) and or:benefit means that this sponsorship package has 3 benefits, i.e., “*conference registration*”, “*link on conference website*”, and “*logo on conference website*”.
- *Event Series* The recurring one-time events shapes an event series, which is represented by the class or:EventSeries. Events within a series usually have a similar name or a common name affix. Members of or:EventSeries class have various object and data-type properties (Fig. 4).

## Events metadata collection and analysis

In this section, we present how event metadata is scrapped from the Web, including event homepages and Twitter account statistics. Furthermore, we present a metadata analysis on top of this data and show which knowledge can be derived from it.

### Data collection

The data collection task is mainly focused on event homepages because they are the main source of information about an event. **Step 1.** Homepages provide unstructured data, therefore the first step is to scrape and clean the data. Further channels were processed while gathering metadata of events, such as crawling WikiCFP, which provides metadata in a well-structured way, and Twitter account statistics. **Step 2.** Store the data in a way that they can be easily processed in large batches and analyzed, i.e., CSV format. **Step 3.** Share the collected data in an accessible way by importing it to *OpenResearch.org* using its bulk import service^19^. Surprisingly, we found that some important conferences do not archive old editions, for example, for the SEMANTiCS conference events are not archived before 2013. The collected data are fully available online through the *OpenResearch.org* platform, which also provides LOD features and lets others further improve and enrich our collected data.

### Data analysis

We create metadata-based metrics to conclude statements about the quality of the considered events and derive conclusions about the scholarly communication of the whole community. The selected metrics have been collected observing successful events as they provide indication for their quality. Due to lack of data, parts of our analysis were not possible for some recent years, such as when studying sponsorship packages for 2020, 2019, and 2018 (see Table 1). In addition, due to the global pandemic occurred in the beginning of 2020, i.e., COVID-19, generally scholarly communication has been affected Subramanya et al. (2020), such as the cancellation of SEMANTiCS 2020, or changes of several events from physical to virtual conferences, such as ESWC 2020. Therefore, some metadata, such as keynote speakers, is not available.

In these analysis, we use four personas to represent the needs and interests of different stakeholders of scientific events. A single metric is not meant to fit all personas at once, but to address different interests and requirements for one or more of the personas. As they address individual requirements for a persona, they are meant as a tool to match events that suit individual needs and interests and not as a global ranking. For each metric, the collected metadata is described first. After that, an analysis of this metric based on some event series is presented to test the collected data. *Sponsors*. One characteristic of events is the existence of sponsors in that event. Event homepages list their sponsors and additional sponsorship opportunities are provided. The latter will be referred to as “sponsor benefits”. Here we will base quality metrics on the willingness of sponsors to pay an amount of money for certain benefits. Events provide so-called “packages” and title them with names like “Gold Sponsorship” or “Bronze Sponsorship”. These packages have different monetary values, for a real-world example, VLDB2017 charges $10,000 for Gold Sponsorship and $3000 for a Bronze Sponsorship. The common benefit classes can be identified such as adding the “logo on the website” or having an “advertisement in conference brochure” which are purchasable at several event series. Events can be compared by their benefits and the minimal price a sponsor must pay to get this benefit. Table 1 shows a list of four conference series with their offered options for a set of benefits over the past six years.

**Table 1:** Some benefits and their minimum price over different events

Benefit	Lowest possible price in the years						
	2017	2016	2015	2014	2013	2012	
Acknowledgement in press releases	6500	6500		6500	7500	6000	ESWC
	3500	4000	4000		3000	4000	ISWC
	4750	3500	3500				SEMANTiCS
	15000	15000	15000				VLDB
Logo on website	500	500	500	500	500	1000	ESWC
	1000	2000	2000	1500	1500	3000	ISWC
	1150	850	850	1750	1750	1750	SEMANTiCS
	3000	3000	3000				VLDB
Booth at conference						3000	ESWC
	7000	7500	7500	2500	4000	5500	ISWC
	4750	3500	3500	2850	2200	2200	SEMANTiCS
	5000						VLDB
Table at exhibition area	3000	3000	3000	3000	3000		ESWC
							ISWC
							SEMANTiCS
	5000		1000				VLDB

**Fig. 5:**
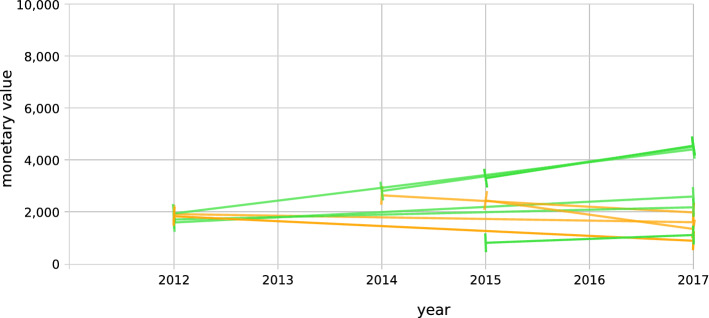
Trend in monetary value of benefits in SEMANTiCS series

Before we compare event series, we look at a single series and how their benefit prices develop over the last six years. Each benefit in a single event series with their price over the years makes a single set of data points. For each set of data points, the gradient was calculated. We group the trend lines by event series and draw the family of trend lines in a single trend chart. For *x* being years and *y* being monitory values, we calculated the gradient *m* of the trend line for *N* data points with the following formula:

$$\begin{aligned} m = \frac{ n \sum {(xy)} - \sum {(x)} \cdot \sum {(y)}}{ n \sum {(x^2)} - (\sum {x})^2 } \end{aligned}$$

In this step, we calculate the intercept *b* with the y axis as

$$\begin{aligned} b = \frac{\sum {(y)} - m \cdot \sum {(x)}}{N} \end{aligned}$$

Hereby, we present the points for a single common benefit per each single event of a series given as a 2D vector. The yearly values are shown in the first dimension and the monetary values are in the second dimension. Figure 5 shows such a trend chart for the SEMANTiCS conference series illustrated for years of 2012 to 2017. In this period, the sponsors could get the following benefit types: Acknowledgment in press releases, free conference registrations, advertise in the conference brochures, advertised via social media, advertisement inside the conference material and proceedings and in participant bags, article on the conference website, banners at the conference venue (physical conferences), booth at the conference, logos appearing at the conference website, logos appearing in the conference brochure, having own workshop or co-occurring events, giving speeches at the conference, adding sub-pages on the website, tweet with specific hashtags, and gaining Twitter followers by the conference iteself or its participants. Each benefit makes a single set of data points. Along the y axis, we have the monetary value of the benefit. As the gradients of the trend lines are not easy to see all the time we colored trend lines with a positive gradient in half opaque green and the ones with a negative gradient in half opaque orange. The trend lines start at the first year the benefit is available and end at the last year the benefit is available. For SEMANTiCS, we overall observed nine positive and five negative trends. The strongest positive gradient of the long-term benefits is of the benefit “booth at the conference” which costs a minimum of 2200€ in 2012 and 4750€ in 2017. The only higher gradient for SEMANTiCS is of “acknowledgment in press releases“ which develops from 2012 with 3500€ to 2017 with 4750€. The two going trends from 2012 to 2017 are “logo on website” and “logo in conference brochure”. They started quite high but reduced the minimal price for the last years to a lower value, which you can also see in Table 1. Another interesting point to see in the trends is that when SEMANTiCS changes from a sister-event as i-SEMANTiCS in 2014 to its own event since 2015 many new benefits come available for sponsors.

**Fig. 6:**
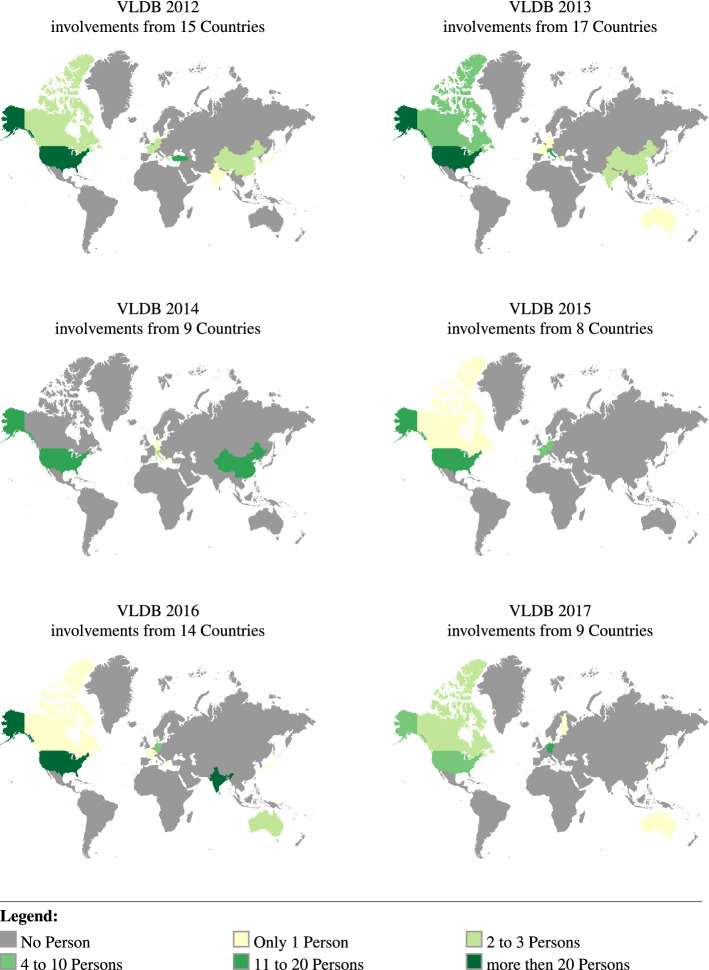
Map illustration for regions of event organizers involved in organizing one of the events in VLDB event series, shown per year

*Organizers origin* The term “origin” is used as the current home location or workplace of the person and not where the person is born. Figure 6 shows the origin of the persons involved in organizing one of the events in the VLDB series from 2012 to 2017.

It can be noticed that, for VLDB there are not many different countries per year, but some countries appear repeatedly for each year, so we queried the data again and this time we count how many events in this period are (by person involved in organizing the event) associated with this country. Table 2 shows the amount of persons for each country in sum from 2012 to 2017. In this case, Canada is only ranked number eight. Italy, which is only associated with two from six events, is in the top five.

**Table 2:** Summed country participation in the number of organizing persons from VLDB2012 to VLDB2017

Order	Country	Amount of persons
#1	USA	112
#2	Germany	28
#3	India	28
#4	China	18
#5	Italy	15
#6	Turkey	12
#7	Switzerland	12
#8	Canada	11
#9	Singapore	10
#10	France	9

**Fig. 7:**
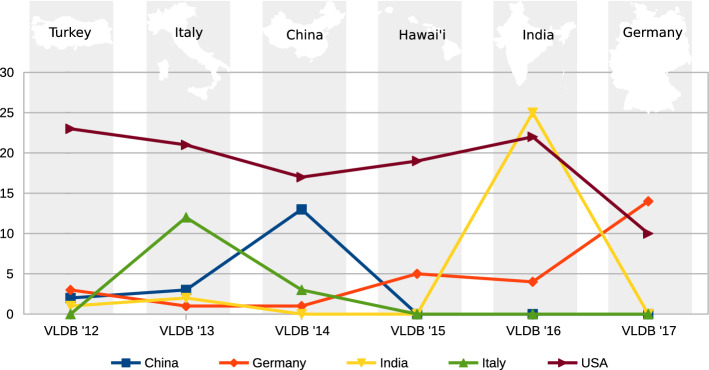
Participation in the VLDB Series from 2012 to 2017, Rank 1 to 5

**Fig. 8:**
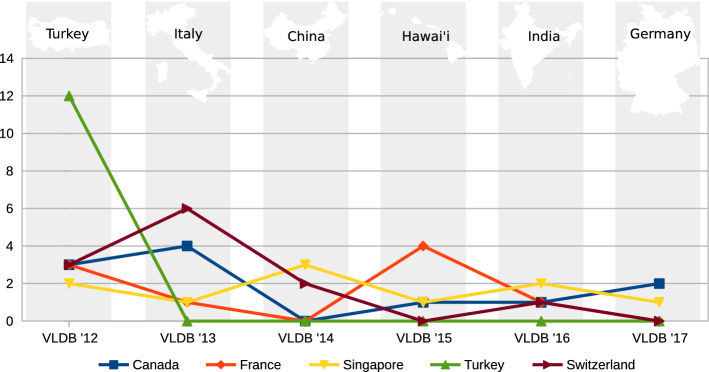
Participation in the VLDB Series from 2012 to 2017, Rank 6 to 10

The key question here is: *Is there a trend for each country over the years?* For readability, we only include the top ten countries and split them into two groups of five. Figures 7 and 8 shows the number of persons from a country over the event series. We observed peaks by a country participating in the organizing of an event whenever the event is located in this country or a neighboring country. For example, Turkey is highly involved in the VLDB event of 2012, and India is highly involved in 2016. It seems that VLDB events use locals for organizing the event if possible.

**Fig. 9:**
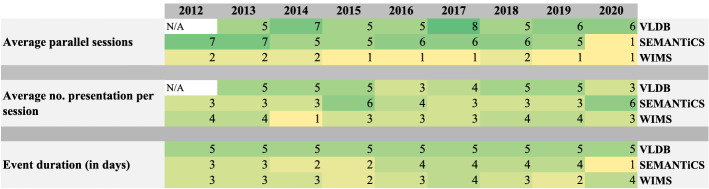
Average numbers of parallel sessions and number of presentations per session for the event series VLDB, SEMANTiCS and WIMS in the last decade

*Event duration* A metric to match events for individual preferences on event duration and program structure can easily be derived from the event start and end date. The event program structure for VLDB, SEMANTiCS, and WIMS have been manually collected, as these data are not available in a structured way across all events in our sample. Figure 9 shows the average number of parallel sessions, the average number of presentations (rounded values) per session, and the event duration for VLDB, SEMANTiCS, and WIMS in the last decade. For VLDB2012, no program information is available, so the cells in the program structure remain empty. Assuming a researcher prefers events with a single track and no parallel sessions. He can use this metric to find matching events, such as the latest WIMS iterations. And if he wants to have multiple parallel sessions, he can schedule the presentations that he wants to attend.

**Fig. 10:**
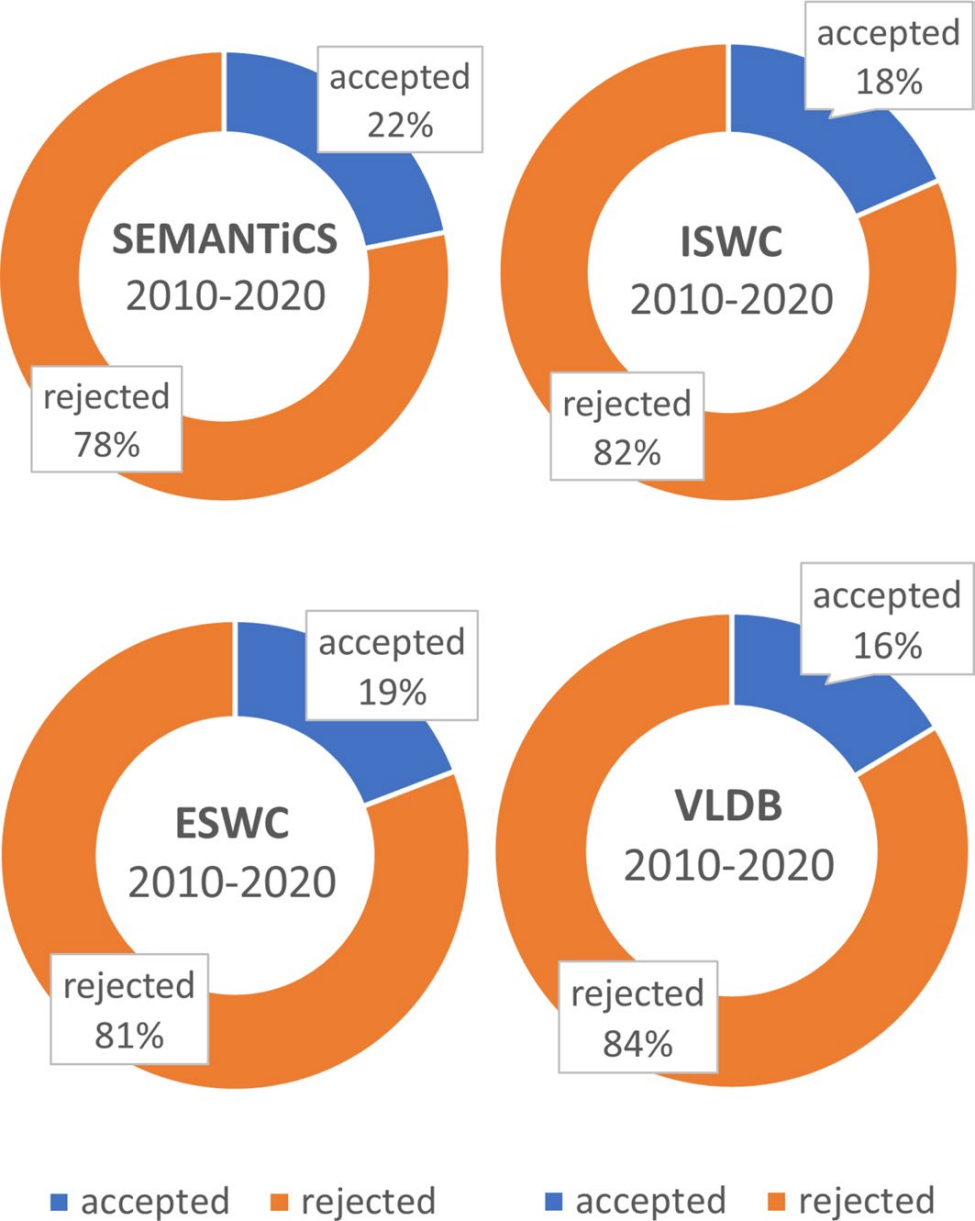
The average number of accepted and rejected papers of SEMANTiCS, ISWC, ESWC and VLDB in the last decade (i.e., 2010–2020)

*Acceptance Rate* The acceptance rate of a conference in a particular year is defined as the ratio between the number of accepted articles and the number of submitted ones. The average acceptance rate (AAR) has been calculated for all editions of a particular series to get an overview of the overall acceptance rate of this series since the beginning. Figure 10 shows the average number of accepted and rejected papers of SEMANTiCS, ISWC, ESWC, and VLDB in the last decade (i.e., 2010–2020).

**Fig. 11:**
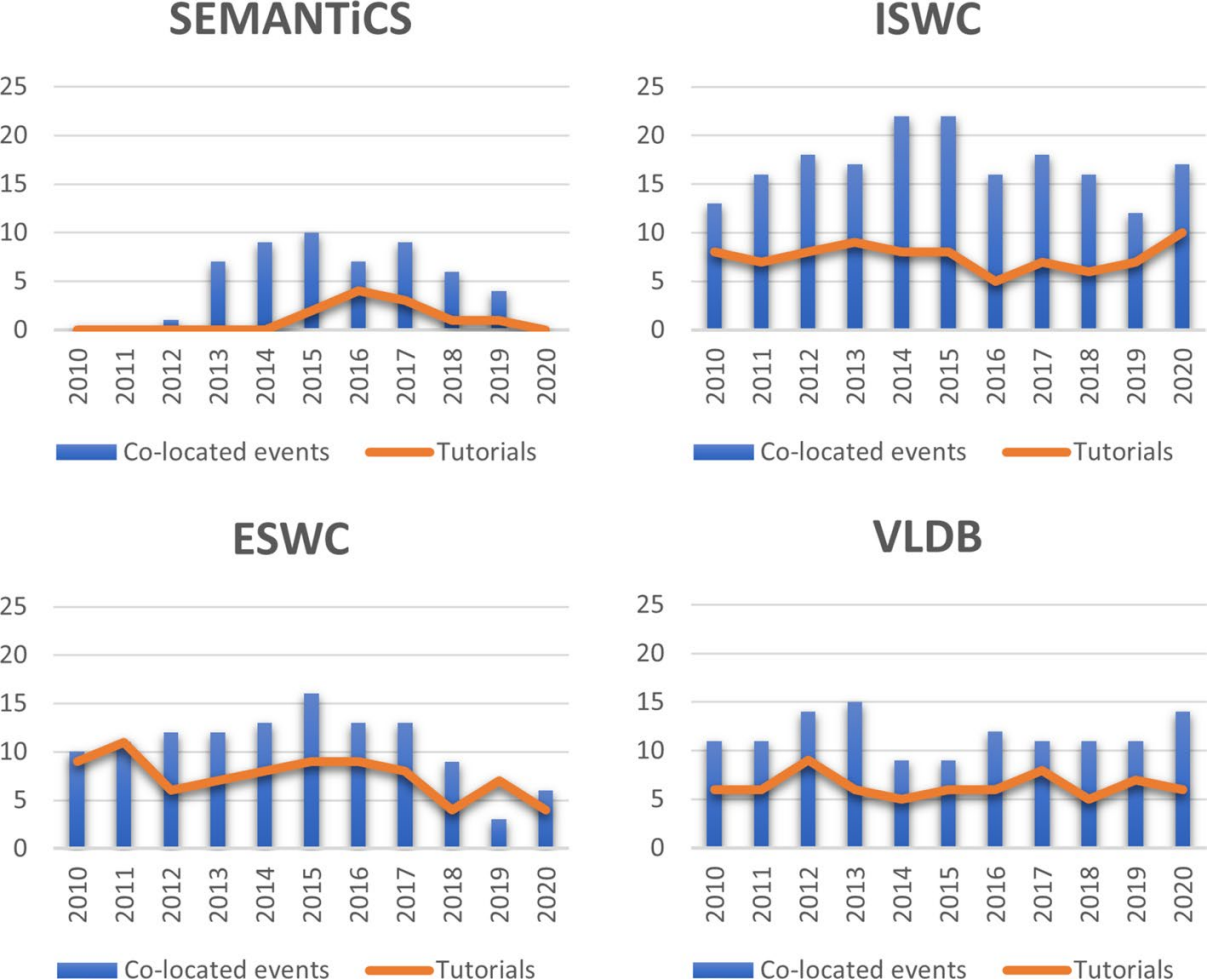
Number of co-located events and tutorials in SEMANTiCS, VLDB, ISWC and ESWC in the past decade (i.e., 2010–2020)

*Events Co-location* Many of the scientific events have co-located events, often categorized as conferences, workshops, tutorials, presentations, or exhibitions. The latter is often connected to a special sponsorship model. We reviewed the co-located events with SEMANTiCS, VLDB, and the years 2012 to 2017. Figure 11 shows the number of co-located events and tutorials in SEMANTiCS, VLDB, ISWC, and ESWC in the period 2010–2020. ISWC has a very strong standing with an average of 17 workshops in the whole period. In comparison, SEMANTiCS has the lowest average of 5 collocated workshops per event.

*Keynote Speaker* All events in our dataset have keynote speeches in their program. Renowned keynote speakers based on their expertise in a special field, accomplishment, or affiliation are an option to raise interest in attending the event. At the moment, to assess the reputation of a scientist, author-level metrics are widely used. These include the widely used h-index Hirsch (2005) or i10 index created by Google Scholar^20^. All authorship statistics for this work are obtained from the respective Google Scholar profiles. Table 3 shows all keynote speakers of SEMANTiCS and ESWC, their affiliation, an average of author-level metrics of all speakers in the period 2012–2020. The collected data in the past seven years shows that some events show a tendency to the industry, while others show a tendency to the academic world, based on the affiliation of keynote speakers. Each individual event of SEMANTiCS has at least three keynote speakers with industrial affiliation. In 2014, there was no keynote speaker from academia at all. Exceptionally, in 2018, speakers from academia exceed the ones from industry. In ESWC, the number of speakers from academia exceeds the number of speakers from the industry in most of the years. On average four keynotes from industry and two from academia could be observed for SEMANTiCS series from 2012 to 2018, while an average of two keynotes from both industry and academia are given at ESWC series in the same period.

**Table 3:** The average h-index and i10 of the keynote speakers at SEMANTiCS and ESWC in the period 2012–2020

	SEMANTiCS				ESWC			
	Industry	Academia	Avg. h	Avg. i10	Industry	Academia	Avg. h	Avg. i10
2010	–	–	–	–	0	4	53	140
2011	–	–	–	–	4	3	84	215
2012	3	2	39	122	5	3	31	127
2013	4	1	49	127	2	2	63	215
2014	4	0	–	–	1	3	41	162
2015	4	2	31	68	0	3	62	108
2016	3	2	10	16	1	2	77	198
2017	3	2	18	36	2	1	48	98
2018	4	6	27	63	1	2	28	58
2019	3	3	31	71	0	3	44	99
2020	–	–	–	–	2	1	51	129

SEMANTiCS in 2020 was cancelled due to COVID-19 pandemic

## Semantic mediaWiki platform

This work is an extension of the initial OpenResearch.org Vahdati et al. (2016) platform which provides a semantic wiki for scholarly artifacts from papers to events. Here we cover certain parts of event ontology that was still missing in the original Openresearch.org. This includes an extensive look into sponsorship of the events. After defining the ontology in general, we present how it can be implemented at OpenResearch.org Vahdati et al. (2016) and what opportunities are given by that. An already implemented wiki system is used as the basis for injecting the defined schema for scientific events. The OpenResearch.org platform is based on Semantic MediaWiki^21^ (SMW). SMW is an extension to MediaWiki^22^, which adds semantic annotations to explicitly state facts which turns a Wiki (with all known Wiki features) into a collaborative database (with all known semantic knowledge graph features, like adding facts and querying the graph).

Semantic MediaWiki extensions advance the internal linking and add semantic meaning to the links. An article about a subject represents the subject itself in SMW and a link from one article to another represents a special relationship between the subjects. In SMW these links can be prefixed with a not displayed *property name*. The OpenResearch.org ontology specifies *or:isFollowedBy* for the relationship between two subsequent events. A reasoner can now identify this relationship and include this fact. If a user queries what is the following event for VLDB2012, the VLDB2013 wiki page will be returned. In addition to semantic linking between articles, Semantic MediaWiki also introduces a similar function to express facts that have a literal data value as an object.

*Templates* Another feature of the MediaWiki that is heavily used by Semantic MediaWiki are Templates^23^ which come in handy to ease the annotation process^24^. If a user simply wants to fill in facts about a subject, the user can use predefined templates in the article page body text. These templates take arguments in a structured way, then they process them and return the markup code for the page.

*Semantic Forms* On top of these templates is another function of SMW, the Page Forms^25^. Page Forms allow defining forms in the wiki which create a single page and fills templates in this page with the values from form elements. These forms give the user the same power as using the template directly, but with a user-friendly interface. For instance, users can add event metadata using the semantic form we created for events.^26^

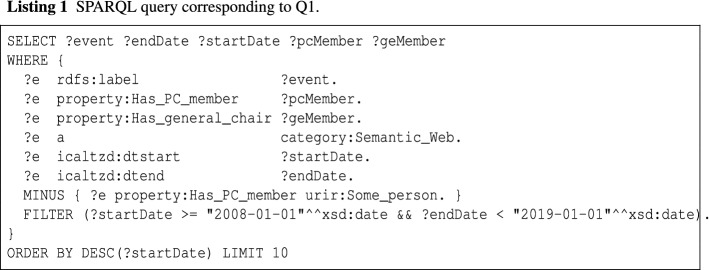



*SPARQL endpoint* OpenResearch.org has its own SPARQL endpoint for querying its RDF dataset. The SPARQL endpoint of OpenResearch.org is available at https://www.openresearch.org/sparql.

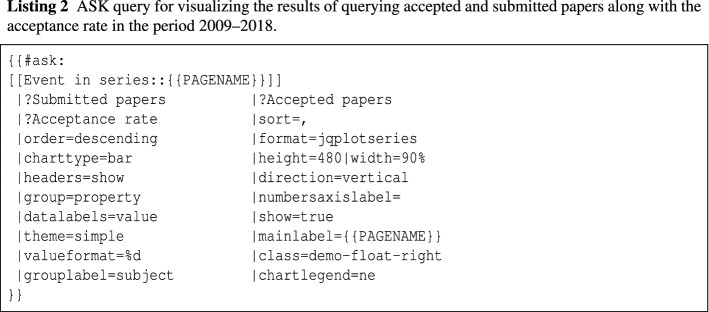



One example of the competency queries that OpenResearch.org can answer is “Q1: *List the PC members and general chairs who were involved in semantic web related events in the last decade*”. Listing 1 shows the corresponding SPARQL query of such query. Currently, a list^27^ of interested queries are presented on OpenResearch.org platform. These queries have been implemented considering several quality metrics. Manual effort on finding the same results of this query from the current systems is costly and time consuming. However, looking at many other communities this is actually what is happening. Many researchers either gain such knowledge over many years and by having an overview of the scientific communication in their discipline, or search through many resources to combine such information and conclude facts for themselves.

**Fig. 12:**
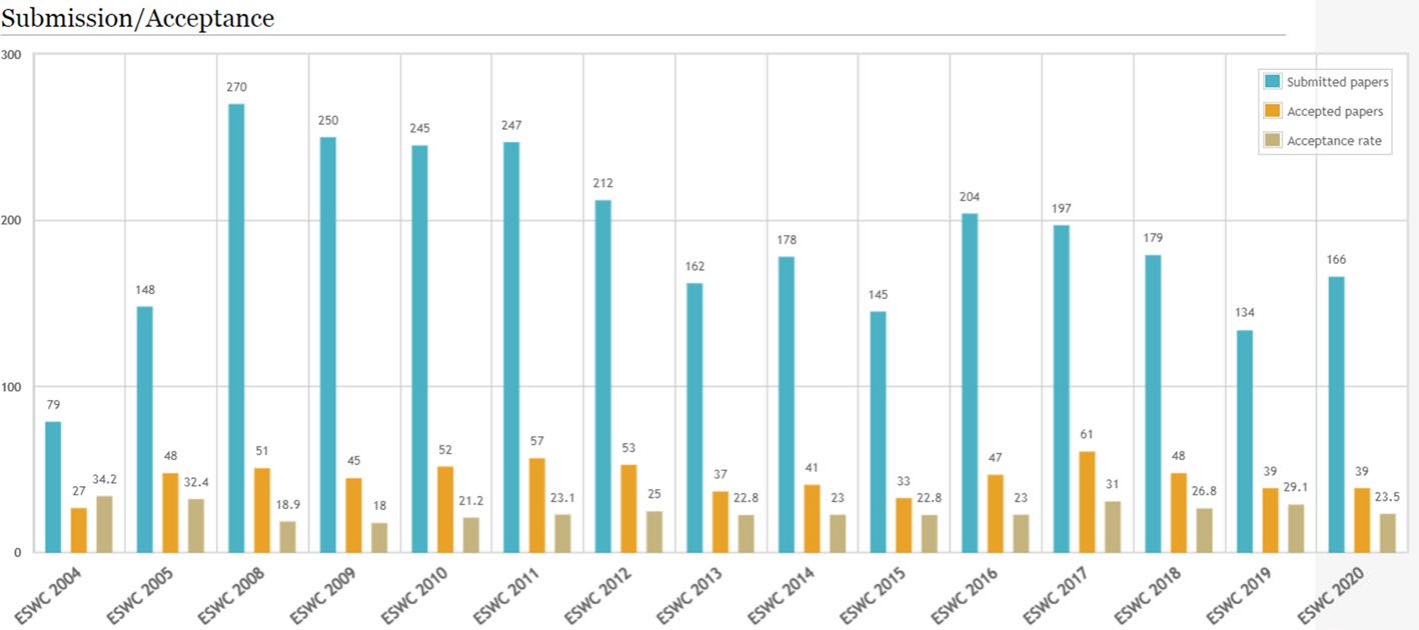
Visualization of query results in OpenResearch.org using SMW extensions

*SMW extensions* The “Semantic Result Formats” is an extension to semantic mediawiki (SMW) that supports a numerous number of further formats in the description of results, including formats for maps, calendars, timelines, charts, graphs, and mathematical functions. The result formats can be used in inline queries and other semantic searches. Listing 2 shows the inline query for visualizing the results (Fig. 12) of querying accepted and submitted papers along with the acceptance rate for the ESWC conference series in the period of 2004 to 2020 using Semantic Result Formats extension in OpenResearch.org.^28^

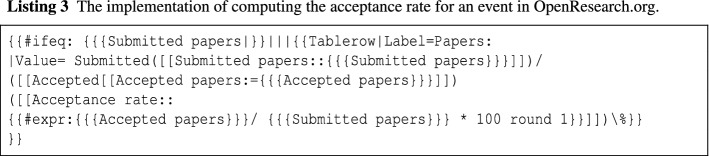



Implementation of the captured metadata in this research is also considered in the OpenResearch.org ontology that has been developed with an on-demand decision-making process. Some of the metrics suited to be defined as raw properties and some others have been computed by queries over the data (using MediaWiki expressions). The implementation of the acceptance rate as a complex metric that can be calculated from the raw properties has been performed in the template of the corresponding event (Listing 3). Note that Openresearch.org is semantic wiki and crowd a sourcing-based system. Although the aim is to improve the foundation of the system by completing its ontology developments and adding visual data analytic features, the main challenge in gathering data. There are several publicity activities in action as well as bulk data import possibility to bridge this gap.

## Conclusion and future work

In this article, we study common characteristics of renowned events by analyzing their metadata. First, we provide a description of the world of scientific events in the context of OpenResearch.org (RQ.1). The ontology of OpenResearch.org, which was already aligned with other ontologies, has been extended by introducing new concepts, such as sponsorship, and a more variable model for the role of event organizers. After defining the concept of scientific events and their properties more clearly, the next driving question was whether events can be compared using these properties (RQ.2). One of the hidden characteristics is the amount of sponsoring that sponsors invest in an event. In this regard, we compared and analysed the sponsoring costs associated with the same benefits across the four conference series. There are notable differences, which hint that well-established, renowned conferences can convert their reputation into increasing sponsorship revenues. We obtained criteria based on event metadata and showed that it is possible to build metrics for these criteria that can be used to compare events (RQ.3). With these metrics, researchers or other stakeholders can compare events and find reasonable matches for their intent. Towards automating the analysis introduced in this work employing the OpenResearch.org platform, our plan is to employ ML-based approaches for generating recommendations.

In the future, we aim to implement all the proposed tools directly plugged into the Openresearch.org platform. The ontology is open for further improvement by different communities as well as its developers. In addition, it is possible to include even more metadata about events (e.g. about keynotes). Another future work direction is a stronger interlinking with other data sets and ontologies. Another future work might be to use the constructed knowledge graph from OpenResearch as a source for knowledge graph analysis techniques and suggest new events based on this knowledge. A major change wrt. organizing and attending scientific events in the year 2020 was due to the global pandemic of COVID-19 virus. Due to preventing health issues many of the gatherings including scientific events and educational activities which were planned as physical gatherings had to change. Some of these changes have created enormous challenges for the organizers as well as attendees and some others brought a step forward towards digitization. As a future work, we plan to analyse the changes and their effect in the research trends.

## Footnotes

1 http://www.openresearch.org.

2 https://cfplist.com/.

3 https://semanticscholar.org.

4 http://conference.city/.

5 http://papersinvited.com/.

6 https://dblp.org/.

7 https://dl.acm.org/proceedings.cfm.

8 https://www.elsevier.com/solutions/scopus.

9 http://ieeexplore.ieee.org/Xplore/home.jsp.

10 http://scigraph.springernature.com/explorer/about/.

11 http://lod.springer.com/data/search.

12 http://scholarlydata.org/dumps/.

13 http://www.wikicfp.com/cfp/.

14 https://www.cfplist.com/.

15 http://confsearch.org.

16 http://dblp.uni-trier.de/db/.

17 http://www.conference.city/.

18 http://www.allconferences.com.

19 A description of how collected CSV data is transformed to a bulk of wiki pages is available at https://www.openresearch.org/wiki/Openresearch:HowTo.

20 While h-index counts the *h* papers with a citation number greater than *h*, i10 counts number of publications with at least 10 citations.

21 https://www.semantic-mediawiki.org/.

22 https://www.mediawiki.org/.

23 https://www.mediawiki.org/wiki/Help:Templates.

24 https://www.semantic-mediawiki.org/wiki/Help:Semantic_templates.

25 https://www.mediawiki.org/wiki/Extension:Page_Forms.

26 https://www.openresearch.org/wiki/Special:FormEdit/Event.

27 https://www.openresearch.org/wiki/Sparql_endpoint/Examples.

28 https://www.openresearch.org/wiki/ESWC.
